# A new spiny frog of the genus *Pristimantis* (Anura, Strabomantidae) from the eastern slopes of the Ecuadorian Andes

**DOI:** 10.3897/zookeys.1269.162260

**Published:** 2026-02-13

**Authors:** Juan Pablo Reyes-Puig, Mario H. Yánez-Muñoz, Santiago R. Ron, Pablo J. Venegas, Jhael Ortega, Julio C. Carrión-Olmedo, Carolina Reyes-Puig

**Affiliations:** 1 Fundación EcoMinga Red de Protección de Bosques Amenazados, Quito, Ecuador Instituto de Biodiversidad Tropical IBIOTROP, Museo de Zoología & Laboratorio de Zoología Terrestre, Colegio de Ciencias Ambientales, Universidad San Francisco de Quito (USFQ) Quito Ecuador; 2 Unidad de Investigación, Instituto Nacional de Biodiversidad (INABIO), Quito, Ecuador Reserva: The Youth Land Trust Washington United States of America; 3 Fundación Oscar Efrén Reyes, Departamento de Ambiente, Baños, Ecuador Maestría en Conservación y Manejo de Biodiversidad, Pontificia Universidad Católica del Ecuador Quito Ecuador; 4 Maestría en Conservación y Manejo de Biodiversidad, Pontificia Universidad Católica del Ecuador, Quito, Ecuador Museo de Zoología QCAZ, Pontificia Universidad Católica del Ecuador Quito Ecuador; 5 Museo de Zoología QCAZ, Pontificia Universidad Católica del Ecuador, Quito, Ecuador Unidad de Investigación, Instituto Nacional de Biodiversidad (INABIO) Quito Ecuador; 6 Curador de Herpetología Instituto Peruano de Herpetología (IPH), Lima, Peru Fundación EcoMinga Red de Protección de Bosques Amenazados Quito Ecuador; 7 Reserva: The Youth Land Trust, Washington DC, USA Fundación Oscar Efrén Reyes, Departamento de Ambiente Baños Ecuador; 8 Smithsonian's National Zoo and Conservation Biology Institute, Center for Species Survival, Front Royal, 22630, USA Curador de Herpetología Instituto Peruano de Herpetología (IPH) Lima Peru; 9 Instituto de Biodiversidad Tropical IBIOTROP, Museo de Zoología & Laboratorio de Zoología Terrestre, Colegio de Ciencias Ambientales, Universidad San Francisco de Quito (USFQ), Quito, Pichincha, Ecuador Smithsonian's National Zoo and Conservation Biology Institute, Center for Species Survival Front Royal United States of America

**Keywords:** Amphibia, Andean slopes, morphology, *Pristimantis gualacenio* species complex, taxonomy

## Abstract

A new species of rain frog from the upper montane forest of the eastern Andes in the upper Pastaza watershed, Ecuador is described. *Pristimantis fergusoni***sp. nov**. is characterized by its short snout, conical tubercles on upper eyelids and heels, combined with a scarlet colored belly in females. These unique traits differentiate the new species among other *Pristimantis* of upper montane eastern Ecuadorian Andes. Phylogenetic analyses support its validity and place it within a clade nested with other tuberculated species occurring along the Andean slopes. *Pristimantis fergusoni***sp. nov**. is currently known from only two close localities (Cerro Candelaria and Chamana Reserves) at elevations between 2972 and 3200 m elevation, within the Llanganates-Sangay Ecological Corridor. Data Deficient IUCN status is proposed until new information is available. Individuals were observed at night perched on vegetation in herbaceous and arbustive vegetation of upper montane forest. This discovery contributes to a growing body of evidence identifying the upper Pastaza valley as a hotspot of amphibian endemism and diversification. With this addition, 30 strabomantid species are described for this region in the last decades, underscoring its conservation importance. This discovery highlights the urgent need for integrated taxonomic efforts combining fieldwork, morphology, and molecular data to resolve complex evolutionary relationships within the megadiverse genus *Pristimantis*, especially in underexplored high-elevation habitats of the tropical Andes. Finally, we provide an updated phylogeny for this clade that clarifies its evolutionary relationships.

## Introduction

Frogs of the genus *Pristimantis* Jiménez de la Espada, 1870, currently comprises 626 species (Frost 2025) and are particularly diverse in the northern Andes of South America (Duellman and Lehr 2009). In Ecuador, the *Pristimantis* highest diversity is found on the eastern slopes of the Andes (Coloma and Duellman 2024). Members of this clade are characterized by polymorphic, polychromatic, and phenotypically plastic patterns, demonstrating to be a taxonomically challenging group (Guayasamin et al. 2015; Yánez-Muñoz et al. 2016; Páez and Ron 2019).

In many regions worldwide, limited scientific funding has historically constrained the widespread use of genetic analyses in taxonomy. As a result, numerous species, including several species of *Pristimantis*, have been described primarily on the basis of morphological evidence (Reyes-Puig and Mancero 2022). Fortunately, during the last decade, an increasing number of integrative studies combining molecular and morphological data have been produced by a growing community of specialist taxonomists in Colombia, Ecuador, and Peru, leading to the discovery and description of dozens of new species (e.g., Urgilés et al. 2019; Ortega et al. 2022; Zumel et al. 2022; Reyes-Puig et al. 2023)

Within the Ecuadorian Andes, several *Pristimantis* species exhibit conspicuous dorsal tubercles that produce a spiny, often greenish appearance, and variation in coloration, tubercle development, and skin-fold patterns provides informative characters for distinguishing among congeners (Yánez-Muñoz et al. 2014; Navarrete et al. 2016). Although these arboreal, spiny-skinned frogs comprise several lineages whose relationships remain poorly resolved due to limited genetic data, current evidence indicates that the spiny phenotype evolved independently multiple times as an ecological adaptation to arboreal vegetation substrates (Reyes-Puig and Yánez-Muñoz 2012; Yánez-Muñoz et al. 2014; Guayasamin et al. 2015; Navarrete et al. 2016).

Recent fieldwork expeditions and herpetological collections, particularly in remote and poorly biologically explored, such as Sangay National Park and its buffer zone continue to uncover previously undescribed species (e.g., Harvey et al. 2013; Brito M. et al. 2016; Navarrete et al. 2016; Brito et al. 2017; Chasiluisa et al. 2020; Ortega et al. 2022). The last herpetological survey of the Llanganates Sangay Corridor (INABIO et al. 2023) revealed several unnamed species. Here, we describe one of them, a new frog of the genus *Pristimantis* based on morphological and molecular data.

## Materials and methods

### Taxonomy and nomenclature

For family classification, we followed the proposal of Hedges et al. (2008) and Heinicke et al. (2018). For new species recognition, we adopted the unified species concept by De Queiroz (2005, 2007). This definition considers new species as independent lineages of metapopulations, separately evolving with particular secondary properties (e.g., reproductive isolation, a distinct ecological niche, a unique combination of character states) that are considered to merit taxonomic recognition as species, with several supporting lines of evidence. We followed morphological definitions, description format, and terminology proposed by Lynch and Duellman (1997) and Duellman and Lehr (2009). For color names descriptions we follow Köhler (2012).

### Fieldwork and specimen management

Fieldwork expeditions campaigns were conducted between 2008 and 2023 in Cerro Candelaria and Chamana Reserve at eastern Ecuadorian Andes by teams of Instituto Nacional de Biodiversidad (**INABIO**), Museo de Zoología of the Pontificia Universidad Católica del Ecuador (**QCAZ**), and Museo de Zoología of the Universidad San Francisco de Quito (**ZSFQ**). Detailed methods of sampling and fieldwork are detailed in MECN et al. (2013) and Brito et al. (2017) and we followed recommendations made by Lips et al. (2001).

Each captured specimen in the field was assigned a unique number and was taken to the base camp in an individual plastic bag to confirm its sex and reproductive condition. We recorded the time and date of capture and other ecological data related to each specimen.

We followed ethical protocols suggested by Beaupre et al. (2004), and the collection and research permits used in this research was provided by the Ecuadorian government (INABIO: MAE-DNB-CM-2019-0120, MAATE-ARSFC-2023-3346, MAATE-ARSFC- 2024-0847; USFQ: MAE-DNB-2018-0106).

### Morphology

To facilitate the identification of dorsal, ventral, and flank patterns, the specimens were photographed with a unique code number and stored in a catalog of photographic references. The specimens collected were euthanized with a benzocaine solution, pinned in 10% formalin, and preserved in 70% ethanol. The sex and reproductive condition of each individual was determined by identification of secondary sexual characteristics (i.e., nuptial pads, males with vocal slits, and body size) and by direct gonad inspection through dorsolateral incisions. The morphometric measures were taken with an electronic caliper (precision ± 0.01 mm, approximated to 0.1 mm) following the scheme of Duellman and Lehr (2009), as follows: **SVL** (snout-vent length), **TL** (tibia length), **FL** (foot length, from the proximal margin of inner metatarsal tubercle to the tip of Toe IV), **HL** (head length, from the angle of the jaw to tip of snout), **HW** (head width, at the level of the angle of the jaw), **ED** (eye diameter), **TD** (tympanum diameter), **IOD** (interorbital distance), **EW** (upper eyelid width), **IND** (internarial distance), and **EN** (eye–nostril distance), **FaL** (Forearm length), **HaL** (Hand Length), **TaL** (Tarsus length). Comparative lengths of toes III and V were determined when both were adpressed against toe IV; lengths of fingers I and II were estimated when pushed towards each other. The life patterns were extracted from field notes and color images.

Coordinates and elevation of the type localities were recorded by a GPS and later validated by ArcGis. Throughout the text, the following abbreviations are used for photo credits and associated information for collected specimens: Mario H. Yánez-Muñoz (MYM), Juan P. Reyes-Puig (JPRP), Bioweb Santiago R. Ron (BioWeb SRR). Finally, we followed the proposal of (Gardenfors et al. 2001), and Ortega-Andrade et al. (2021) at a national scale to estimate the threat of extinction risk.

### Revised material at scientific collections

Morphological comparisons were based on the examination of museum specimens, including species closely related to the new species described herein (*Pristimantis bellae* Reyes-Puig & Yánez-Muñoz, 2012; *P. gualacenio* Urgilés, Sánchez-Nivicela, Nieves & Yánez-Muñoz, 2014; *P. eriphus* Lynch & Duellman, 1980; *P. incanus* Lynch & Duellman, 1980; *P. roni* Yánez-Munoz, Bejarano-Munoz, Brito-M. & Batallas-R., 2014; *P. katoptroides* Flores, 1988; *P. inusitatus* Lynch & Duellman, 1980; and, *P. galdi* Jiménez de la Espada, 1870). Topotypic material of these species was also studied (see Appendix 1). All examined specimens are deposited in the Herpetology Collection of the Instituto Nacional de Biodiversidad (**INABIO, DHMECN**), the Zoology Museum of the Pontificia Universidad Católica del Ecuador (**QCAZ**), and the Zoology Museum of the Universidad San Francisco de Quito (**ZSFQ**).

### Laboratory analyses

Laboratory work took place at two laboratories in Quito: The Nucleic Acid Sequencing Laboratory of the Instituto Nacional de Biodiversidad-INABIO and the Molecular Laboratory of the Zoology Museum at QCAZ. At INABIO, DNA was extracted from the liver or muscle under standard protocols using GeneJET Genomic DNA Purification Kit (K0722). We targeted a partial mitogenome flanking 12S rRNA to the ND1 region. Polymerase Chain Reaction parameters are described in Reyes-Puig et al. (2024) and Yánez-Muñoz et al. (2025). Sequencing run was performed on a minION mk1c using Flongle Flow Cells R10.4.1 and Rapid Barcoding Kit 96 V14 (SQK-RBK114.96) following manufacturer protocols. Raw reads were super accurate (SUP) basecalled and demultiplexed with Dorado 7.6.7. Consensus sequences and downstream bioinformatics followed the protocol described in Yánez-Muñoz et al. (2025). At QCAZ, we followed laboratory procedures described in Ron et al. (2020). Sequencing was conducted by Macrogen Inc. (Seoul, Korea). Raw sequences (forward and reverse) were assembled in Geneious Prime 2020 (https://www.geneious.com) using de novo mapping.

### Phylogenetic analyses

We performed a BLASTn search using the 16S rRNA fragment to include closely related lineages in an exploratory phylogeny. BLASTn hits were retained only when they met the following minimum similarity thresholds: ≥ 85% sequence identity and ≥ 60% query coverage. Subsequently, we refined the sampling by incorporating additional species that were not retrieved in the BLASTn results, likely due to short query sequence length, we performed a new BLASTn search using a custom database of unpublished sequences from INABIO genetic bank. The sampling was also expanded based on other tuberculated species distributed on the eastern Ecuadorian Andes (*Pristimantis bellae* and *Pristimantis* sp.) with available sequences on INABIO and QCAZ genetic banks. To ensure the replicability of our results, the DNA matrix is available in Zenodo (https://doi.org/10.5281/zenodo.17792775).

We included sequences from the subgenus *Hypodictyon* as outgroups based on Hedges et al. (2008). Sequences were originally published by Heinicke et al. (2007); Zhang et al. (2013); Hutter and Guayasamin (2015); and Sánchez-Nivicela et al. (2020).

We built a DNA character matrix in Mesquite (Maddison and Maddison 2025) with nine newly generated sequences and 64 sequences retrieved from GenBank. We constructed a partitioned matrix with the partial mitogenomes. The partitions were as follows: 12S rRNA, tRNA-Val, 16S rRNA, tRNA-Leu, and codon positions in ND1. Matrices were aligned using default parameters in MAFFT (Katoh and Standley 2013) and visually inspected for unambiguous alignment errors.

Substitution models and Maximum Likelihood Tree Inference were performed using IQ-TREE (Trifinopoulos et al. 2016) under default settings. Branch support was evaluated using 5000 ultrafast bootstrapping and SH-aLRT tests with 5000 replicates (Guindon et al. 2010). Support values mentioned herein follow this format: SH-aLRT support (%)/ultrafast bootstrap support (%). Uncorrected p-distances were calculated with an 800 bp-long fragment of 16S rRNA using MEGA 11 (Tamura et al. 2021).

The partial mitogenome matrix comprises 3742 bp for 82 individuals. IQTree best substitution models for seven partitions are the following: TIM2+F+I+G4 for 12S rRNA, HKY+F+G4 for tRNA-Val, TIM2+F+I+G4 for 16S rRNA, K2P+I for tRNA-Leu, K2P+G4 for ND1 first position, TPM3+F+I for ND1 second position, and TN+F+G4 for ND1 third position.

## Results

### Systematics

**Figure 1.**
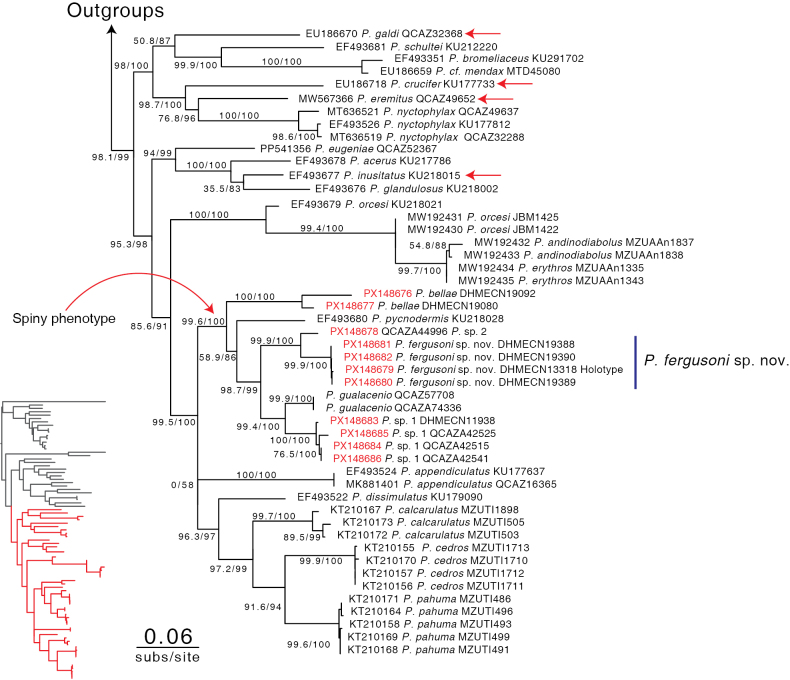
Phylogenetic relationships of *Pristimantis fergusoni* sp. nov. and its congeners based on mtDNA sequence data. Terminals highlighted in red correspond to sequences generated in this study. Outgroups are shown at the top and in the inset (left); the inset also illustrates the full tree with the focal clade highlighted in red. Numbers at nodes indicate branch support values following this format: SH-aLRT support (%)/ultrafast bootstrap support (%). Scale bar: 0.06 substitutions per site. GenBank accession numbers are shown to the left of the species name; number of voucher museum specimen are shown to the right. Red arrows show species with spiny phenotype.

Our molecular phylogenetic analysis (Fig. 1, Table 1) provides strong support for the new species, with branch support values of 99.9/100. This species forms a clade (99.9/100) with another undescribed species from Sangay National Park in Morona province, referred to herein as *P.* sp. 2. This clade is in turn sister to the clade (99.4/100) formed by *P. gualacenio* and another undescribed species, *P.* sp. 1 from Sangay National Park in Morona province. Together, these two clades form a group that is sister to *P. pycnodermis* Lynch, 1979 with low branch support (58.6/86), while *P. bellae* is recovered as the sister taxon to all of the aforementioned species (99.6/100).

**Table 1.** Genetic p-distances between *Pristimantis fergusoni* sp. nov. and its closest congeners.

	*Pristimantis bellae*	*Pristimantis pycnodermis*	*Pristimantis* sp. 2	*Pristimantis fergusoni* sp. nov.	*Pristimantis gualacenio*
*Pristimantis bellae*					
*Pristimantis pycnodermis*	9.84%				
*Pristimantis* sp. 2	10.57%	6.55%			
*Pristimantis fergusoni* sp. nov.	11.07%	7.51%	2.80%		
*Pristimantis gualacenio*	8.33%	6.37%	5.96%	6.81%	
*Pristimantis* sp. 1	10.64%	6.19%	7.41%	7.59%	3.59%

#### Pristimantis fergusoni sp. nov.

Taxon classificationAnimaliaAnuraCraugastoridae

6137E4E9-B56D-591A-A8DA-F27E0C3F94E0

https://zoobank.org/8C0982B1-55A6-468E-BDC9-C6AAAC749357

**Figure 2.**
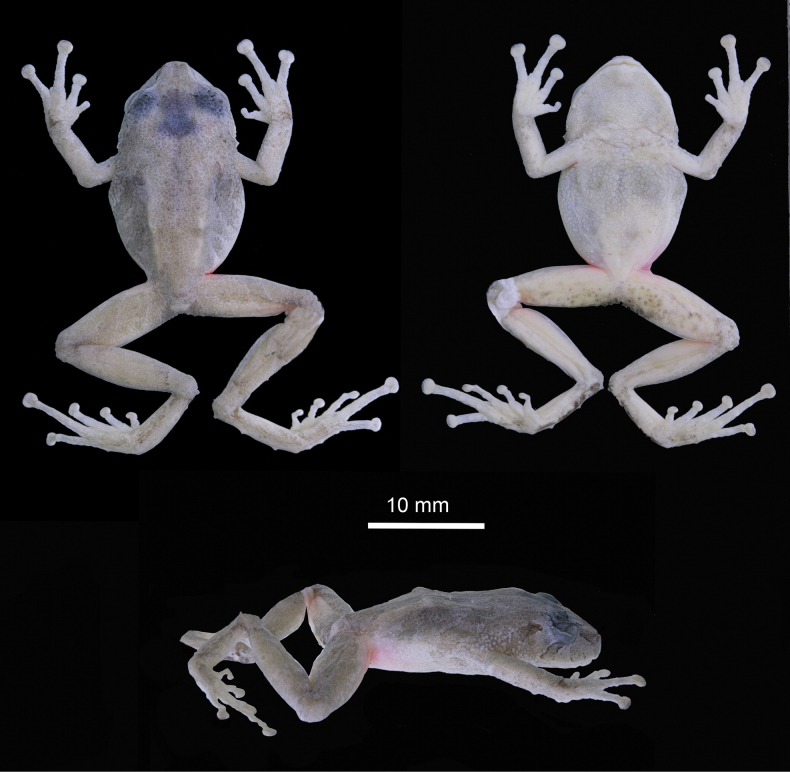
Dorsal, ventral, and lateral view of the holotype (DHMECN 13318) of *Pristimantis fergusoni* sp. nov. in preservative. Photographs by JPRP.

**Figure 3.**
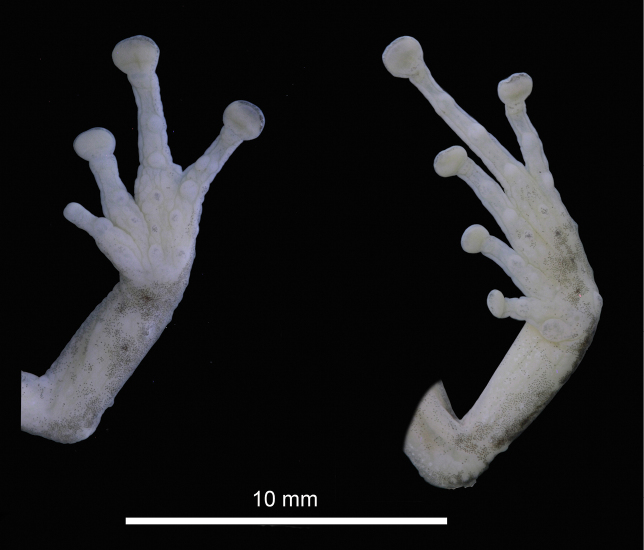
Palmar and plantar view of the holotype (DHMECN 13318) of *Pristimantis fergusoni* sp. nov. in preservative. Photographs by JPRP.

**Figure 4.**
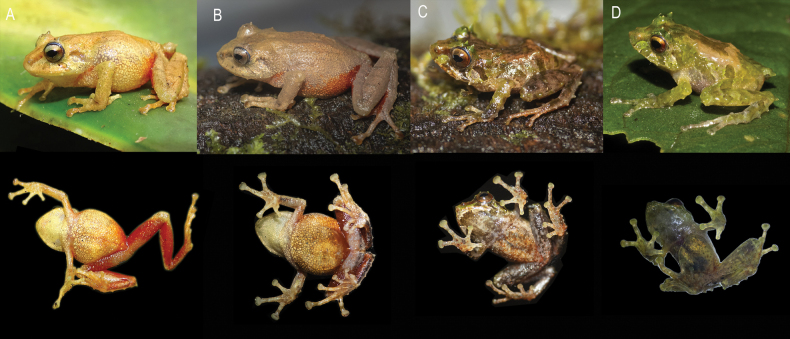
Life color and sexual dimorphism in *Pristimantis fergusoni* sp. nov. **A**. Female holotype DHMECN 13318; **B**. Female DHMECN 19390; **C**. Male DHMECN 19388; **D**. Male DHMECN 19389. Dorsal and ventral view, respectively. Photographs by JPRP.

**Figure 5.**
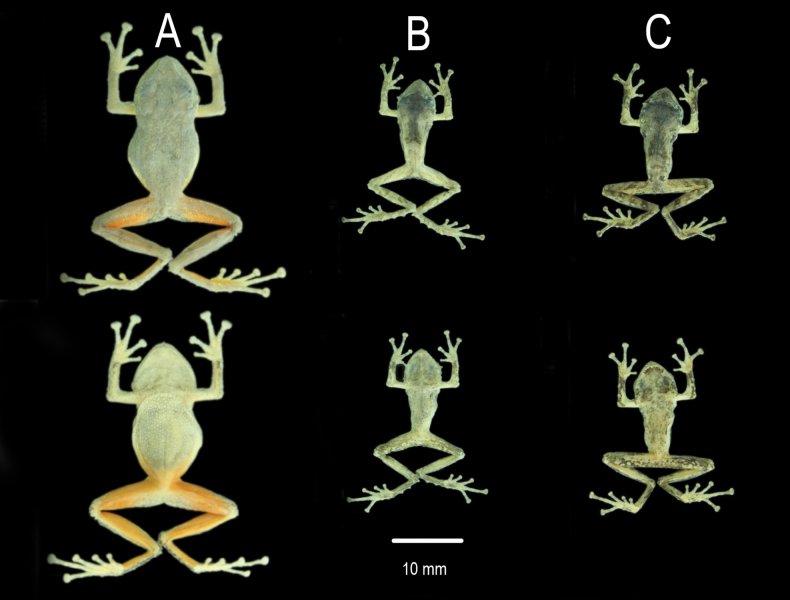
Dorsal and ventral color pattern variation in *Pristimantis fergusoni* sp. nov. in preservative Paratypes. **A**. Female DHMECN 19390; **B**. Male DHMECN 19388; **C**. Male DHMECN 19389. Photographs by Gabriela Lagla.

**Figure 6.**
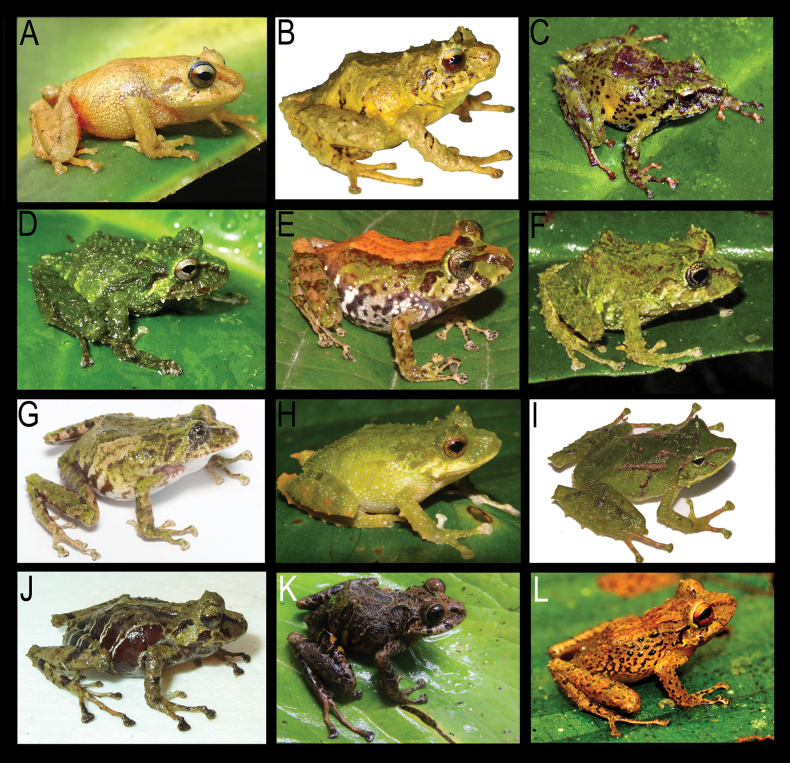
Live photographs of the new species and comparison with related and similar *Pristimantis* species from eastern Andes of Ecuador. **A**. *Pristimantis fergusoni* sp. nov., female holotype DHMECN 13318, from Cerro Candelaria; **B**. *P. gualacenio* DHMECN 10748, from Area de Conservación Municipal Tinajillas; **C**. *P. bellae* DHMECN 4812, from Cerro Candelaria; **D**. *P. eriphus* DHMECN 5209, from Río Zuñag; **E**. *P. incanus* DHMECN 11857, from Reventador; **F**. *P. roni*, DHMECN 11313, from Sardinayacu; **G**. *P. katoptroides* QCAZ58900, from Sadinayacu; **H**. *P. inusitatus* not collected, from Napo San Isidro; **I**. *P. galdi* DHMECN 9640, from Reserva Tapichalaca; **J**. *P. colonensis* DHMECN 6414, from La Bonita; **K**. *P. venegasi* MZUTI 6571; **L**. *P. yanezi* DHMECN 13309, from Río Zuñag. Photographs by JPRP, MYM, SRR, Juan Carlos Sánchez-Nivicela, and Patricia Bejarano-Muñoz.

**Figure 7.**
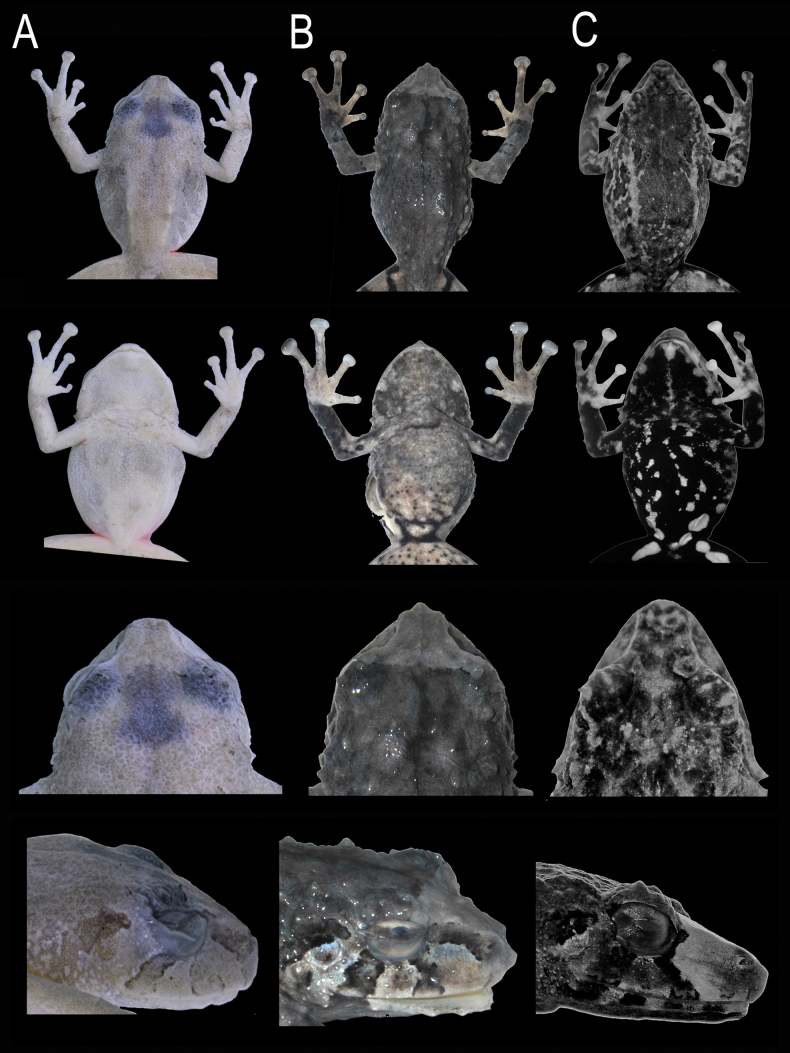
Preserved comparison of three spiny frogs from eastern Ecuadorian slopes: dorsal, ventral, and snout profiles in detail. **A**. *Pristimantis fergusoni*, female holotype DHMECN 13318; **B**. *Pristimantis gualacenio*, holotype DHMECN 10747; **C**. *Pristimantis bellae*, female holotype DHMECN 4812. Photographs by JPRP & MYM.

Figs 2, 3, 4, 5, 6, 7 Proposed standard English name: Ferguson’s Robber Frog Proposed standard Spanish name: Cutín de Ferguson

Pristimantis sp. nov. O INABIO et al. 2023: 41, fig. 166.

##### Generic placement.

The new species is assigned within the genus *Pristimantis* based in our phylogeny and by having head as wide as body, tympanic membrane, and cranial crests usually differentiated, dentigerous process of vomer present, condition of the adductor muscle “S”, terminal discs on digits expanded, Toe V as long or longer than Toe III, skin on dorsum and venter variable (Heinicke et al. 2007).

##### Type material.

***Holotype***. • DHMECN 13318, adult female (Figs 2, 3, 5, 6), collected by Juan Pablo Reyes-Puig, Esteban Morales, and Luis Recalde, at Cerro Candelaria Reserve, Rio Verde, Baños, Tungurahua, Ecuador (1.463851°S, 78.302931°W; 3210 m elevation) on 28 December 2016. ***Paratypes***: • (14 total; 8 ♂/ 6♀; Figs 3, 4). Ecuador: DHMECN 13317 and DHMECN 13320 adult females collected in the same locality and collection data of the holotype • DHMECN 19390, adult female collected in the same locality and collection data of the holotype by Juan Pablo Reyes Puig and Esteban Morales on 25 July 2025 • QCAZ 58581, QCAZ 58584, adult females collected by Juan Pablo Reyes-Puig, at Cerro Candelaria Reserve (1.462184°S, 78.303139°W; 3200 m elevation) on 25 January 2015 • QCAZ 58582, QCAZ 58583, QCAZ 58585 and QCAZ 58586 adult males collected in the same day and by same collectors than QCAZ 58581 • DHMECN 19388 and 19389, males collected at the same locality and data collectors than DHMECN 19390 • DHMECN 16176, male collected at the same locality of the holotype by Juan Pablo Reyes Puig on 11 November 2019; ZSFQ 3874; adult male collected by Carolina Reyes-Puig, Juan Pablo Reyes Puig, David Brito-Zapata, Lía Altamirano, Mateo Toral, Sebastián Ramos, Yelena Macías, Alejandra Romero, Larissa, Samay Lima and Max Cardenas at Chamana Reserve, Baños, Ulba, Tungurahua (1.42616°S, 78.39572°W; 3015 m elevation) on 7 November 2019.

##### Diagnosis.

A new species of *Pristimantis* which can be distinguished by the following unique combination of morphological characters: (1) SVL 18–22.9 mm in females (*n* = 6) and 12.2–16.4 mm in males (*n* = 8); (2) skin on dorsum smooth to slightly shagreen with scattered subconical tubercles, more prominent on the head and scapular region in the males, inconspicuous occipital fold present or not; skin on flanks with flattened tubercles and ventrolateral region areolate; skin on venter coarsely areolate with scattered enlarged warts, more evident on males; discoidal fold absent; low dorsolateral folds present, or at least evident in the first half of body; (3) tympanic membrane and tympanic annulus present, its upper and posterolateral margin covered by supratympanic fold; (4) snout short, subacuminate in dorsal view, rounded in profile bearing a pointed papillae on tip (especially distinct in males); (5) upper eyelid with small tubercles and one or three conical and enlarged tubercles; eyelid width less (65%) than interorbital distance IOD; cranial crests absent; (6) dentigerous processes of vomers absent: (7) males with vocal slits and nuptial pads slightly evident; (8) Finger I shorter than Finger II; discs of digits expanded, slightly truncate apically; (9) fingers bearing distinct narrow lateral fringes; (10) ulnar tubercles present, conical and distinct or weakly defined; tarsal tubercles present (two or four) conical, distinct or low; (11) heel with one or two prominent conical tubercles; outer edge of the tarsus with one or two conical tubercles; inner tarsal fold present; (12) inner metatarsal tubercle elliptical, distinct, two times larger than outer; outer metatarsal tubercle small, ovoid; numerous distinct supernumerary plantar tubercles, low; (13) toes with distinct narrow lateral fringes; basal toe webbing absent; Toe V slightly longer than Toe III (tip of the disc on Toe V scarcely reaching the proximal border of distal subarticular tubercle on Toe IV); toe discs about as large as those on fingers; (14) in life, dorsal pattern range from Light Chrome Orange, Light Orange Yellow, or Lime Green to Cinnamon, with or without Tawny marks in the interorbital area and scapular region; Scarlet flash coloration in the hidden surfaces of posterior limbs and groins (more accentuated in females, males have pale Scarlet tones with Dusky Brown); ventral surface Cream Yellow to Brownish Olive with scarlet coloration on venter and thighs. Shanks, tarsus, palms, and soles with Scarlet tones; iris Spectrum yellow with a median Burnt Orange streak.

##### Comparative diagnosis.

Detailed comparisons between *Pristimantis fergusoni* sp. nov. and other morphologically similar spiny *Pristimantis* species from the eastern slopes of the Ecuadorian Andes are summarized in Table 2, Figs 6, 7. *Pristimantis fergusoni* sp. nov. is most similar to *Pristimantis gualacenio* which possesses subconical, flattened tubercles on the upper eyelid, a rounded snout, rounded tubercles on the heel, black shanks, and hidden surfaces with a dark background and yellow or orange bands. Additionally, *Pristimantis gualacenio* exhibits dentigerous processes of the vomers (Urgilés et al. 2014). In contrast, *P. fergusoni* sp. nov., has one to three enlarged, conical tubercles on the upper eyelid; one or two prominent subconical tubercles on the heel; Scarlet shanks and hidden surfaces; and lacks vomerine teeth.

**Table 2.** Comparison of diagnostic coloration and morphological characters in the new species and most similar spiny frogs of eastern versant of Ecuadorian Andes.

Species	Iris color	Dorsal color patterns	Ventral color patterns	Groin and hidden surfaces of the thigh	Upper eyelid tubercle	Tubercles on the heels	Dorsolateral folds	Dorsal and lateral view of the snout	SVL (mm) in males	SVL (mm) in females
*Pristimantis fergusoni* sp. nov.	Light Orange Yellow with a Burnt Orange horizontal stripe	Light Chrome Orange, Lime Green to Cinnamon in several tones	Cream Yellow to Brownish Olive with Scarlet coloration on posterior region; males with pale scarlet tones and Dusky Brown	Scarlet flash coloration, more evident in females; In males pale scarlet tones with Dusky Brown pigmentation	Pungent conical surrounded by smaller subconical tubercles	Subconical	Present, low evident on the first half of the body	Subacuminate dorsal and lateral l view bearing a pointed papilla on the tip (especially distinct in males)	13.6–19.11	16.31–23.89
*Pristimantis gualacenio* Urgilés et al., 2014	Coppery with a horizontal red stripe	Dark brown to light reddish brown	Dark reddish brown, with light gray marks.	Black with yellow or orange	Two subconical and several flattened tubercles	Rounded and flattened	Absent	Rounded	16.8	22.2–24.7
*Pristimantis bellae* Reyes Puig & Yánez Muñoz, 2012	Coppery golden	Brown with green tones, reddish Brown	Black with white marks in females, males gray mottled with dark and pale marks	Black with white marks in females, gray with dark and pale marks in males	Prominent, conical	Prominent, conical	Present, rugose	Subacuminate and rounded with small papillae	22.1–23.9	25.8
*Pristimantis yanezi* Navarrete et al., 2016	Coppery with a reddish horizontal stripe.	Olive green with X-shaped or rhomboidal dark brown mark on scapular region, scattered brown flecks or blotches	Ventral surfaces of shanks dirty white or white with dark brown diagonal stripes	Groins white or tan with distinct black or dark brown diagonal stripe	Low conical tubercle and some low indistinct tubercles posteriorly	Two or three low conical tubercles	Absent	Rounded	24.0–27.0	29.8
*Pristimantis eriphus* Lynch & Duellman, 1980	Bronze heavily flecked with black	Brown above with indistinct darker brown chevrons	Brown with diffuse brown spots	Posterior surfaces of thigh barred white and dark brown; white spots on concealed shank	Conical tubercle	Small conical tubercles	Absent	Rounded	18.1–25.2	25.8–29.0
*Pristimantis incanus* Lynch & Duellman, 1980	Dull bronze to gray with a median horizontal reddish-brown streak	Green	Posterior thigh rich brown	Venter gray in most, dull brown or dirty cream with brown flecks in some individuals	Not enlarged	Tubercles not enlarged	Absent	Subacuminate to round in dorsal view, round. in lateral profile	47.3–52.0	46.7–49.6
*Pristimantis roni* Yanez-Munoz et al., 2014	Copper brown with black reticulations	Green	Greenish cream speckled with grey spots in females, greyish belly with white markings on spots	Greenish cream in females, reddish yellow in males	One large conical tubercle and two to five subconical tubercles	Two to three large conical tubercles	Absent	Rounded	14.6–17.5	24.2–28.5
*Pristimantis galdi* Jiménez de la Espada, 1870	Green	Bright green with or without white, gold, orange, red, or brown fringes	White with solid white dots and black flecks usually in the throat	Posterior region of thighs green	One conical tubercle	Conical tubercles present	Present, formed by rows of tubercles	Acuminate in dorsal view and truncate in lateral view	17.1–24.8	28.1–34.0

Another species in the region with conical tubercles on both the upper eyelid and heel is *Pristimantis bellae*. However, it differs from *P. fergusoni* sp. nov. by having black coloration with white markings on the hidden surfaces of the groins and thighs (Scarlet in the new species; Fig. 6).

*Pristimantis yanezi* Navarrete, Venegas & Ron, 2016, *P. galdi*, and *P. inusitatus* do not exhibit flash coloration on the groins. Conversely, *P. eriphus* Lynch & Duellman, 1980, *P. colonensis* Mueses-Cisneros, 2007, *P. incanus*, *P. roni*, and *P. venegasi* Ortega, Brito & Ron, 2022 display white or yellow spots on the groins (whereas *P. fergusoni* exhibits pale Scarlet coloration and iris Spectrum yellow with a median Burnt Orange streak; Fig. 6).

Other species from the region that show red coloration on the groin include *Pristimantis tungurahua* Reyes-Puig, Yánez-Muñoz, Cisneros-Heredia & Ramírez, 2010, which differs by having a protruding snout and red coloration on both the groin and axilla (in contrast, *P. fergusoni* sp. nov. has a short, subacuminate snout in dorsal view with a pointed papilla at the tip, and Scarlet red coloration restricted to the groin). *Pristimantis burtoniorum* Reyes-Puig, Reyes-Puig, Franco-Mena, Jost & Yánez-Muñoz, 2022 shows darker red tones on the groin and has three or four subconical tubercles on the upper eyelid (vs Scarlet groin coloration and 1–3 enlarged conical tubercles in *P. fergusoni* sp. nov.); and finally, *P. sacharuna* Reyes-Puig, Reyes-Puig, Pérez-Lara and Yánez-Muñoz, 2015, which lacks dorsolateral folds and exhibits a single subconical tubercle on the upper eyelid (in contrast to weakly evident dorsolateral folds and 1–3 enlarged conical tubercles in the new species).

##### Description of the holotype.

Adult female (DHMECN 13318; Figs 2, 3) with a robust body and slender limbs; head narrow, not as wide as body, slightly wider than long; head width 40% of SVL; head length 37% of SVL; snout short, subacuminate in dorsal view and rounded in lateral view with a point papilla on the tip; eye–nostril distance 104% of eye diameter; nostrils elliptical, directed laterally; canthus rostralis curved in dorsal view, straight in profile; loreal region concave; lips rounded; upper eyelid bearing low diffuse tubercles with one enlarged conical tubercle; upper eyelid width 65.5% of IOD; tympanic annulus present, barely defined, with dorsolateral margin covered by supratympanic fold; tympanic membrane present, distinct; tympanum diameter 52% of eye diameter, tympanum–eye distance 58.3% of tympanum diameter; one enlarged postrictal tubercle present. Choanae small, ovoid, not concealed by palatal shelf of maxilla; dentigerous processes of vomers absent; tongue slightly longer than wide, not attached in the posterior region, free posteriorly for 2/3 of its length.

Skin on dorsum smooth without scattered tubercles; low dorsolateral fold, evident on that first part of the body; skin on flanks areolate and tuberculated; skin on chest and throat finely areolate and on venter strongly areolate, ventral surfaces of thighs weakly areolate; discoidal fold indistinct; cloacal sheath short; skin in cloacal region tuberculate. Ulnar tubercles present, triangular and low; palmar tubercles present, weakly defined, outer palmar tubercle indistinct, flattened, thenar tubercle present also indistinct; subarticular and hyperdistal tubercles flattened, weakly defined, rounded in ventral and lateral view; small supernumerary tubercles present at the base of fingers, indistinct; fingers bearing distinct lateral narrow fringes; Finger I shorter than Finger II; discs on Finger I, narrow and round, disc on Finger II slightly dilated and round, disc on Fingers III and IV, dilated and slightly truncate; ventral pads on fingers well defined by circumferential grooves on all fingers (Fig. 3).

Hindlimbs slender, tibia length 55.5% of SVL; foot length 50.5% of SVL; dorsal surfaces of hind limbs tuberculate; posterior and ventral surfaces of thighs areolate; knee bearing one distinct conical tubercle; heel bearing one conical tubercle; outer surface of tarsus bearing few low conical tubercles; inner tarsal fold present; inner metatarsal tubercle distinct, elliptical, rounded, two times the size of low, oval, rounded outer metatarsal tubercle; plantar surface tuberculate; subarticular and hyperdistal tubercles distinct, low, rounded in ventral and lateral view; toes bearing distinct narrow lateral fringes; webbing between toes absent; discs nearly as large as those on fingers, most prominent on Toe IV; discs on Toes I, II, and III dilated and rounded, discs on Toes IV and V expanded, weakly truncate; toes having ventral pads well defined by circumferential grooves; relative lengths of toes: I<II<III<V< IV (Fig. 3); Toe V slightly longer than Toe III (disc on Toe III not reaching distal subarticular tubercle on Toe IV, tip of the disc on Toe V scarcely reaching the proximal border of distal subarticular tubercle on Toe IV).

##### Measurements of the holotype (in mm).

SVL 20; TL 11.1; FL 10.1; HL 7.4; HW 8; ED 2.3; TD 1.2; IOD 2.9; EW 1.9; IND 1.7; EN 2.4; FaL: 4.2; Hal; 5.2 TaL:5.12 .

##### Color of holotype in life.

(Figs 4A, 6A) Dorsal surfaces are Light Orange Yellow (77). Presence of canthal, supratympanic and two subocular wide bands Light Chrome Orange (76), dorsal forelimbs and hind limbs are Light Orange Yellow (77), concealed surfaces of the groin and posterior surfaces of the shanks Scarlet (69), ventral surfaces Cream Yellow (82) and posterior region of the venter and groins Scarlet. Iris Spectrum Yellow (79) with Burnt Orange (10) medium stripe.

##### Color of holotype in preservative.

(Figs 2, 3) In ethanol, the dorsal surfaces and flanks are Pale Buff (1) banded with, canthus rostralis, upper eyelids, supratympanic fold, and dorsolateral folds Cinnamon (21)brown; ventral surfaces and groins Pale Buff (1) with darker tones, areas with Scarlet color in life became without pigmentation and pale in preservative. The throat, chest, venter, ventral surface of forelimbs, and thighs are brownish cream, palms and ventral surfaces of shanks Light Buff (2), and ventral surface of tarsus with Dark Brownish Olive (127) pigmentation.

##### Variation.

(Figs 4, 5) Variation of the type series measurements is presented in Table 3. Coloration in life: head, dorsum, flanks and dorsal surface of limbs varies from Light Orange Yellow (77), Light Chrome Orange (76), Lime Green (105) to Cinnamon (21) tones; head with or without Tawny (69) or Lime Green (105) dark brown interorbital stripe, lips bars and supratympanic stripe; flanks with Dark Brownish Olive (127) diagonal stripes, groin coloration is pale Scarlet with Brownish Olive (127) or Dusky Brown (285).

**Table 3.** Measurements (in mm) of the type series of *Pristimantis fergusoni* sp. nov. Ranges are followed by means and standard deviations in parentheses. Abbreviations on methods section.

Trait	Females (*n* = 6)	Males (*n* = 8)
SVL	16.61–25.41 (20.66 ± 2.92)	12.23–16.41 (14.23 ± 1.28)
TL	9.08–13.61 (11.56 ± 1.77)	5.66–9.46 (7.56 ± 1.22)
FL	7.74–16.64 (11.31 ± 2.58)	5.42–8.52 (6.85 ± 0.92)
HL	6.29–8.63 (7.58 ± 0.86)	4.90–6.40 (5.59 ± 0.56)
HW	6.51–10.15 (7.87 ± 1.17)	4.76–6.44 (5.39 ± 0.51)
EW	1.72–2.60 (2.07 ± 0.29)	1.30–1.80 (1.53 ± 0.17)
IOD	2.06–3.12 (2.68 ± 0.32)	1.46–2.39 (1.94 ± 0.29)
IND	1.08–2.44 (1.99 ± 0.42)	1.19–1.79 (1.61 ± 0.19)
EN	1.73–2.52 (2.05 ± 0.27)	0.88–1.78 (1.36 ± 0.27)
ED	1.62–2.83 (2.38 ± 0.37)	1.46–2.08 (1.83 ± 0.21)
TD	0.68–1.35 (1.02 ± 0.24)	0.39–0.82 (0.68 ± 0.14)
HaL	5.23–8.12 (7.15 ± 1.20)	3.96–5.52 (4.79 ± 0.57)

Dorsal coloration in preserved specimens is Pale Buff (1), Light Buff (1), and Cinnamon Drab (50) with or without Dark Drab (45) marks like interorbital bar, V-shaped scapular mark, one chevron in the middle of dorsum, and brown or diffuse brown diagonal stripes on the flanks. Ventral coloration in females is usually uniform Pale Buff (1), and in males is variable with Dark Draff (45) coloration on the throat and Burnt Umber (48) mottling or reticulations.

Dorsal skin texture varies between sexes in *Pristimantis fergusoni* sp. nov. Males usually have a more tuberculate dorsum than females, with a distinct interorbital fold and a conical tubercle between the tip of the snout and the interorbital fold. Additional conical tubercles are present behind the eyelids and on the scapular region, sometimes forming a V-shaped scapular fold. In contrast, females have a shagreen dorsum with scattered, less pronounced tubercles.

##### Distribution and natural history observations.

**Figure 8.**
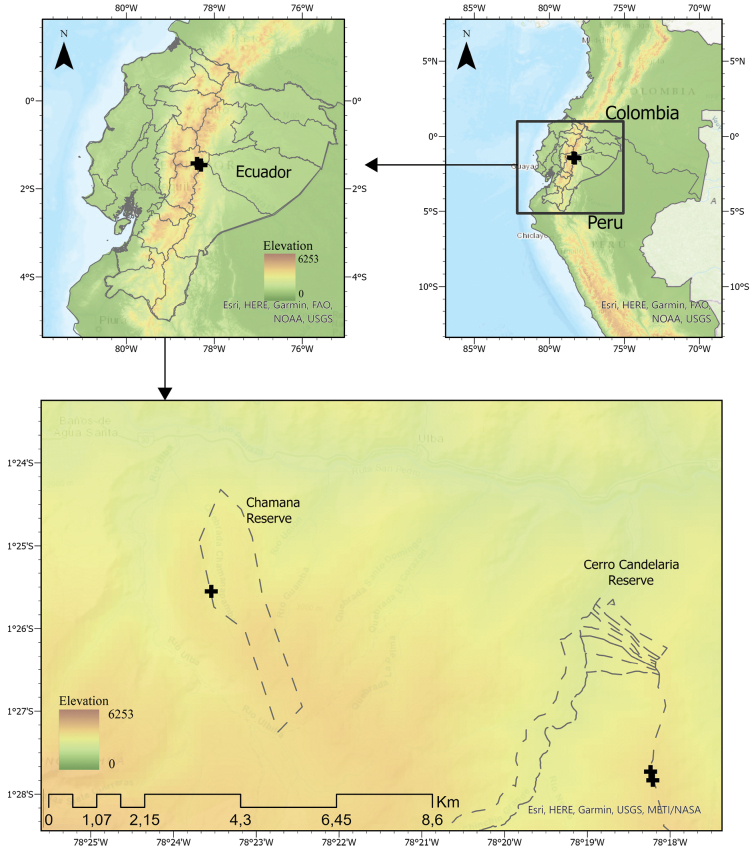
Geographical location of *Pristimantis fergusoni* sp. nov. on the eastern slopes of the Ecuadorian Andes. The right panel shows the position of Ecuador within northwestern South America. The middle panel highlights the distribution of the new species in the country. The lower panel details the known localities within the Chamana and Cerro Candelaria reserves, near Baños de Agua Santa, Tungurahua Province. Elevation is represented by a color gradient. Dashed lines indicate the boundaries of the reserves, and elevation is represented by a color gradient. Map by Carolina Reyes-Puig.

*Pristimantis fergusoni* sp. nov. is known only from two localities within less than 10 km between Cerro Candelaria and Chamana Reserves at elevations of 2972 and 3210 meters, southern to the Pastaza River, in the east-central Andes of Ecuador (Fig. 8). The type locality is located in the Llanganates Sangay Connectivity Corridor, Tungurahua province. The general habitat of the new species is Andean Forest and herbaceous paramo; all individuals were collected during the night, most of them while sitting on leaves of bushes and herbs from 30–140 cm above the ground, mainly in Poaceae (*Neurolepis aristata*, *Cortadeira*) and less in ferns. At Cerro Candelaria the new species was found sympatrically with *Pristimantis puruscafeum* Reyes-Puig et al., 2015, *P. donnelsoni*, and a undescribed *Pristimantis* related to the *devillei* species group (Reyes-Puig et al. 2022).

##### Conservation status.

To assess the current extinction risk of this new species we used the International Union for Conservation of Nature (IUCN) criteria (Gardenfors et al. 2001). *Pristimantis fergusoni* sp. nov. is known from two localities: Cerro Candelaria and Chamana within the cloud forest in the mountains south of Pastaza trench. All known localities are within protected private areas; however, it is likely that other populations may be distributed outside these limits, where land use change is one of the main threats (Gaglio et al. 2017; Jaramillo Coronel et al. 2025). However, until new information is available, we suggest *P. fergusoni* sp. nov. should be categorized as Data Deficient DD with an estimated extent of occurrence < 5000 km^2^.

##### Etymology.

The new species is named in honor of Robert T. Ferguson II, an amateur naturalist and conservationist passionate about herpetofauna worldwide. A citizen scientist, Ferguson helped multiple herp atlas projects in the United States and the conservation and land acquisition in biodiversity hotspots across Latin America. He worked to raise awareness and support for conservation through photography and outreach with Rainforest Trust, Ecominga, and other organizations. This species’ legacy is meant to serve as a reminder that everyone can do something for the betterment of our planet.

## Discussion

With the recognition of the new taxon, 30 strabomantid frogs have now been described on eastern slopes of the Andes in central Ecuador and upper Pastaza Valley in the last decades, reinforcing the hypothesis that the central Andes of Ecuador represent a concentrated center of endemism and diversification for the frogs and certainly for overall biodiversity (Jost 2004; Reyes-Puig et al. 2022; INABIO et al. 2023).

*Pristimantis fergusoni* sp. nov. is supported by both morphological and genetic evidence, reinforcing the view that tuberculated green (“spiny”) *Pristimantis* constitute a polyphyletic assemblage (Fig. 1), as suggested by previous phylogenetic studies (Hedges et al. 2008; Padial et al. 2014; Heinicke et al. 2018), and consistent with broader species-delimitation frameworks and recent systematic work in the genus (De Queiroz 2007; Yánez-Muñoz et al. 2016; Páez and Ron 2019; Reyes-Puig et al. 2024).

We observed 12 species from the montane forests of the eastern Andes of Ecuador, that share spiny skin appearance and distinctive hidden colorations with the new species (*Pristimantis bellae*, *P. colonensis*, *P. eriphus*, *P. galdi*, *P. gualacenio*, *P. incanus*, *P. inusitatus*, *P. katoptroides*, *P. llanganates*, *P. roni*, *P. venegasi*, and *P. yanezi*), but not closely genetic related (Urgilés et al. 2014; Yánez-Muñoz et al. 2014; Navarrete et al. 2016, Ortega et al. 2022). However, *P. fergusoni* sp. nov. is the only species with a scarlet belly (Fig. 6) extending into the upper high montane forest south of the Pastaza trench.

Biogeographically, the clade containing the new species is primarily distributed across the central and southern portions of the eastern slopes of the Ecuadorian Andes. *Pristimantis bellae* is restricted to the central Andes within the Llanganates–Sangay Corridor; *P. pycnodermis* extends farther south into Sangay National Park; and the lineage comprising *P. fergusoni* sp. nov., *P. gualacenio*, and two undescribed species occurs in the montane regions south of the Pastaza River, extending toward the Gualaceo area in southeastern Ecuador

## Supplementary Material

XML Treatment for Pristimantis fergusoni

## Additional information

### Use of AI

In order to improve English we use some translation tools like DeepL Translate.

### Author ORCIDs

Juan Pablo Reyes-Puig https://orcid.org/0000-0002-0730-9356

Mario H. Yánez-Muñoz https://orcid.org/0000-0003-3224-1987

Santiago R. Ron https://orcid.org/0000-0001-6300-9350

Pablo J. Venegas https://orcid.org/0000-0002-6501-4492

Jhael Ortega https://orcid.org/0009-0006-8919-1211

Julio C. Carrión-Olmedo https://orcid.org/0000-0002-4536-6052

Carolina Reyes-Puig https://orcid.org/0000-0001-7828-8698

### Data availability

All of the data that support the findings of this study are available in the main text.

## Appendix 1

**Specimens examined**:

*Pristimantis bellae*: DHMECN 04812, DHMECN 14424, DHMECN 19080

*Pristimantis. eriphus*: DHMECN 11893–11894, DHMECN 13365, DHMECN1371

*Pristimantis fergusoni* sp. nov.: DHMECN 13317–13320, DHMECN 19388–19389 QCAZ 58581–58586.ZSFQ 3874.

*Pristimantis gualacenio*: DHMECN 10747–10756

*Pristimantis incanus*: DHMECN 18498, DHMECN 11585–11597

*Pristimantis galdi*: DHMECN 16803–16804, DHMECN 5195, DHMECN 5607

*Pristimantis inusitatus*: DHMECN 11799, DHMECN 13359,

*Pristimantis katoptroides*: DHMECN 15843, DHMECN 16235,

*Pristimantis roni*: DHMECN 11313–11323
